# *Neisseria meningitidis* Serogroup Y Sequence Type 1466 and Urogenital Infections

**DOI:** 10.3201/eid3101.240940

**Published:** 2025-01

**Authors:** Sebastiaan J. van Hal, Thomas Le, Frances Jenkins, Ratan L. Kundu, E. Athena Limnios, Lucy A. McNamara, Shalabh Sharma, Ellen N. Kersh, Monica M. Lahra

**Affiliations:** University of Sydney, Sydney, New South Wales, Australia (S.J. van Hal); Royal Prince Alfred Hospital, Sydney (S.J. van Hal, T. Le, F. Jenkins); World Health Organization Collaborating Centre for STI and AMR at Prince of Wales Hospital, Randwick, New South Wales, Australia (R.L. Kundu, E.A. Limnios, M.M. Lahra); Centers for Disease Control and Prevention, Atlanta, Georgia, USA (L.A. McNamara, S. Sharma, E.N. Kersh); World Health Organization Collaborating Centre for STI Surveillance at Centers for Disease Control and Prevention, Atlanta (E.N. Kersh); The University of New South Wales, Sydney (M.M. Lahra)

**Keywords:** *Keywords: Neisseria meningitidis*, *bacteria*, *meningitis/encephalitis*, *Neisseria gonorrhoeae*, *urogenital infections*, *niche adaptation*, *Australia*, *United States*

## Abstract

*Neisseria meningitidis* is a common commensal bacterium of the nasopharynx that can cause invasive meningococcal disease (IMD). In comparison, *N. gonorrhoeae* is always a pathogen usually limited to mucosal sites. However, increased evidence for overlapping clinical syndromes is emerging. We compared *N.* *meningitidis* samples from a urogenital outbreak in Australia with sequences from the United States and other countries. We conducted phylogenetic analyses to assess relatedness and examine for genomic changes associated with meningococcal adaptation; we collated a total of 255 serogroup Y (MenY), sequence type (ST) 1466 isolate assemblies. Most urogenital isolates originated from Australia; those isolates formed a distinct clade, most closely related genomically to recent US IMD isolates. No specific genomic changes suggested niche adaptation or associated clinical manifestations. The MenY ST1466 *N.* *meningitidis* isolates circulating in Australia and the United States are capable of causing both urethritis and invasive meningococcal disease.

*Neisseria meningitidis* and *N. gonorrhoeae* characteristically occupy distinct niches in the human body despite evolving from a common ancestor (*1*). *N. meningitidis* bacteria often inhabit the nasopharynx of humans as a commensal bacteria and are generally unencapsulated, or nongroupable. Rarely, meningococci cause invasive disease, such as meningitis or bloodstream infection; those strains are typically encapsulated, or serogroupable (*2*). In contrast, *N. gonorrhoeae* is always considered a pathogen and most commonly infects the mucosa of the pharynx or the anorectal and urogenital tract via sexual transmission (*3*). However, the preferential site of infection may not be as absolute as once thought; overlapping clinical syndromes include urogenital mucosal colonization and local infection caused by *N. meningitidis* (*4*).

The first *N. meningitidis* urogenital infection was documented in 1942 (*5*). Although previous cases had been reported, they were considered a secondary manifestation of invasive disease rather than a de novo urogenital infection. Since then, numerous reports of meningococci causing urogenital infections have been published (*4*). Clinical manifestations are indistinguishable from gonococcal infections; symptomatic infection mainly occurs as urethritis. However, the true prevalence of urogenital meningococcal infections is difficult to determine because current diagnostic testing relies largely on molecular assays targeting *N. gonorrhoeae*. In settings where cultures are performed, laboratory practice for identifying and reporting *N. meningitidis* from urogenital sites varies widely; some laboratories consider those isolates nonsignificant. Similarly, in settings where Gram stains from urogenital samples are used to direct therapy, the presence of gram-negative diplococci would not differentiate between *N. meningitidis* and *N. gonorrhoeae*.

The route of transmission for meningococcal urogenital infections remains unclear. Oral sex is considered a primary likely mechanism (*4*); however, that transmission route for *N. meningitidis* is inefficient and may account for the low observed colonization rates of 1%–3%, with higher rates in men who have sex with men (MSM). Those dynamics may explain why meningococcal urethritis has historically been uncommon.

One exception is the ongoing expansion of a specific clade of nongroupable *N. meningitidis* (US_NmUC), which emerged in 2015 and caused unprecedented clusters of urogenital infections in the United States affecting primarily Black heterosexual men (*6*). Closely related isolates have since been detected in several other countries, including the United Kingdom and Japan (*7*,*8*). The success of the clade is thought to be partly caused by the insertion of IS1301 within the *cps* locus, leading to the deletion of the capsular biosynthesis genes, resulting in a nongroupable phenotype, and by the replacement of the meningococcal *NorB-AniA* denitrification apparatus with one of gonococcal origin, resulting in the capacity to adapt and survive in the urogenital tract (*6*).

Initially limited to the United States, the clade has now been documented in other countries (*9*). In addition to urogenital disease, 7 invasive cases caused by this strain have been reported (*10*); however, they have occurred predominantly in patients with a known or suspected immunocompromising condition, as is typical for invasive meningococcal disease cases caused by nongroupable meningococci (*11*). Regardless, this mainfestation is of concern because it indicates that urogenital *N. meningitidis* infections can be a reservoir for strains that cause invasive disease.

In 2023 in Sydney, New South Wales, Australia, a cluster of symptomatic, urogenital infections with *N. meningitidis* serogroup Y (MenY) sequence type (ST) 1466 (MenY ST1466) was detected. Unlike the US_NmUC, MenY ST1466 has been recently reported to be causing a large number of invasive meningococcal disease (IMD) cases across the United States (*12*). In collaboration with the US Centers for Disease Control and Prevention, we collated the largest publicly available MenY ST1466 genomic dataset to investigate whether the urogenital isolates from Australia represent either sporadic infections or a new emergent niche adaptation event.

## Methods

### Isolate Collection and Sequencing

The isolates included in this analysis were all *N. meningitidis* isolates detected from urogenital samples sent for culture from patients attending sexual health clinics and general practice clinics in New South Wales who were referred to the *Neisseria* reference laboratory (Randwick, New South Wales, Australia) during July–December 2023 (*13*). As is standard procedure for all referred *N. meningitidis* isolates from clinical specimens, the isolates had confirmation of organism identification, antimicrobial susceptibility testing using Clinical Laboratory Standards Institute (CLSI) methodology and breakpoints for penicillin, ceftriaxone, ciprofloxacin, and rifampin, and serogrouping by PCR. All were MenY. By January 1, 2024, a total of 30 urogenital MenY *N. meningitidis* isolates had been referred. We identified 1 additional isolate from October 2019 in the database and included it for sequencing (n = 31).

We performed DNA extraction for 1 colony for each isolate using the EZ1 Advanced XL kit (QIAGEN, https://www.qiagen.com). We generated DNA libraries using an Illumina DNA prep kit (Illumina, https://www.illumina.com) and performed sequencing on the Illumina MiSeq platform according to the manufacturer’s instructions, aiming for a target sequencing depth >20× and Phred quality score >30 across 90% of the fastp version 0.22.0 trimmed reads (*14*). We then generated assemblies using Spades version 3.15.3 and filtered all contigs <1,000 bp long (*15*). We performed in silico multilocus sequence typing (MLST) using BLASTn through PubMLST (*16*); all isolates were ST1466.

### Data Supplementation and Analysis

We supplemented the genomic data collected with additional MenY ST1466 *N. meningitidis* assemblies, including 9 assemblies provided by US CDC representing isolates collected through national invasive meningococcal disease surveillance, as previously described (*17*), and associated with an increase in meningococcal disease among persons with HIV as reported in September 2023 (*17*). We also included all available MenY ST1466 *N. meningitidis* assemblies from PubMLST (n = 224) for which collection year was available (*16*). We reconfirmed the MenY serogroup using characterize_neisseria_capsule (*18*) and the ST1466 group using MLST tools before including isolates in the analysis.

To circumvent inaccurate phylogenetic relationships when using a mapping approach, especially when a very divergent reference is used, we performed long-read sequencing using ONT Nanopore (Oxford Nanopore Technologies, https://nanoporetech.com) to close one of the Australia sequences. We chose the isolate on the basis of the highest N50 assembly metrics from the short-read sequencing data. We obtained a complete circular chromosome of 2.18 Mb (RPAH23R75L) using a hybrid assembly approach implemented through unicycler version 0.4.8 (*19*).

We mapped all assemblies against the generated reference using SNIPPY version 4.0.2 (*20*) specifying the contigs option. We identified recombination using Gubbins version 3.2.1 (*21*). To limit the effect of long branch lengths secondary to missing data, we used the masked alignment to regenerate a phylogeny implemented through IQ-TREE version 2.2.0.3 under a general time-reversible model (*22*). We obtained a time tree through BactDating (*23*) specifying year only and performed geospatial analysis using Nextstrain (*24*).

To explore a possible niche transition, we employed 2 different approaches. First, we compared sequences to the US_NmUC clone (PubMLST identification 47233). Second, we constructed a pangenome using Panaroo version 1.3.4 (*25*) after annotation with Prokka version 1.14.6 (*26*) on all MenY ST1466 assemblies for which the source of infections (i.e., urethritis versus invasive disease) was known. We sought candidate genes for a possible niche change using Scoary version 1.6.16 (*27*), specifying the source as the trait of interest. We performed single-nucleotide polymorphism (SNP) analysis using R package ape version 5.7–1 (*28*). We uploaded sequence reads for all the New South Wales urogenital isolates to the National Center for Biotechnology Information database (project no. PRJNA1117957) (Appendix Table).

## Results

### Isolate Collection

An ongoing outbreak of urogenital MenY began in New South Wales, Australia, in 2023; clinical and epidemiologic data of the first 41 patients was published in 2024 (*13*). Of those cases, 31 urogenital MenY isolates underwent whole-genome sequencing, all from symptomatic patients. All isolates were fully susceptible to penicillin, ceftriaxone, ciprofloxacin, and rifampin; no resistance genes were detected. Phylogenetic analysis of the Australia sequences demonstrated a limited diversity, a median of 17 SNPs (interquartile range [IQR] 8–25) difference among all isolates, suggestive of a clonal expansion event.

**Figure 1:**
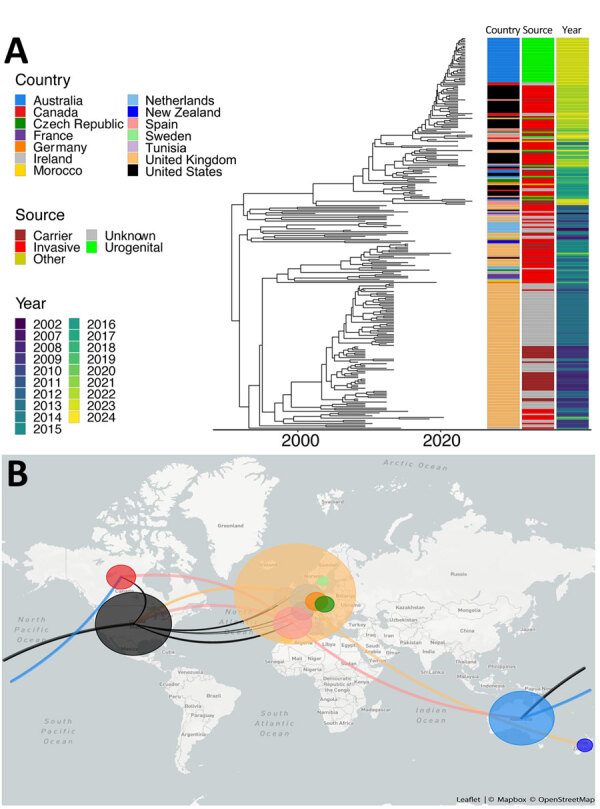
Relatedness of *Neisseria meningitidis* serogroup Y ST1466 isolates from Australia and the United States compared with isolates from other countries. A) Timed maximum-likelihood phylogeny of included isolates of serogroup Y ST1466. Associated metadata are shown to the right of the tree. B) Genomic epidemiology of ST1466 showing transmission lines generated using Nextstrain (*24*). ST, sequence type.

To place those isolates into a global context, we collated a total of 255 MenY ST1466 isolate assemblies, including the urethritis isolates from Australia, invasive isolate sequences shared by the US CDC, and all available MenY ST-1466 sequences in PubMLST (Appendix Table); isolates from the United States, United Kingdom, and Australia represented 80% of the entire dataset that originated from 14 countries. The reported clinical manifestation was absent for 32% of isolates; 36% represented invasive disease and 15% carriage isolates. Most (33/37 [90%]) urogenital isolates originated from Australia (Appendix Table). To circumvent the influence of a suboptimal reference, 1 current outbreak isolate underwent long-read sequencing using ONT nanopore with a single chromosome, RPAH23R75L, generated according to a hybrid assembly approach consisting of 2.18 Mb. Mapping all assemblies to that reference chromosome, the phylogenetic tree after recombination masking (Figure 1, panel A) revealed that most of the Australia isolates formed a distinct clade. Two Australia isolates from 2019 and July 2023, before the urethritis outbreak began in Australia later in July 2023, were intermixed with the US sequences. Those isolates were distinct from the outbreak isolates.

**Figure 2:**
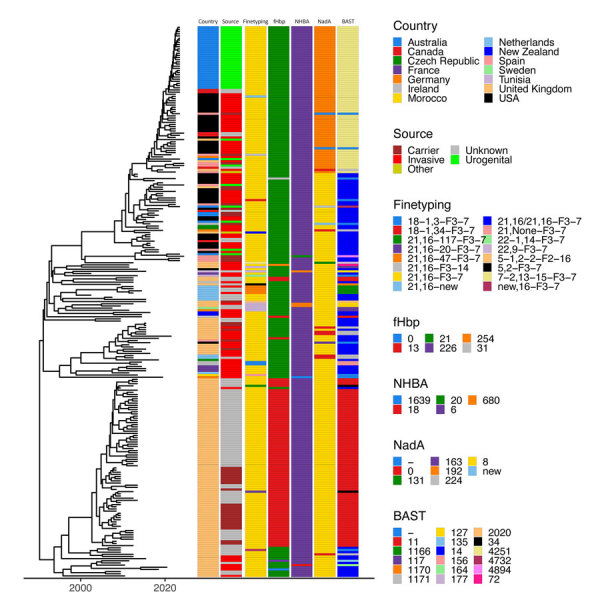
Timed maximum-likelihood phylogeny showing finetyping of *Neisseria meningitidis* serogroup Y ST1466 isolates from Australia and the United States compared with isolates from other countries. Associated metadata shown to the right of the tree are country of origin; source; finetyping profile; virulence profiles for fHbp, NHBA, NadA allele types; and overall BAST sequence typing result. Dashes indicate insufficient or incomplete data. BAST, Bexsero Antigen Sequence Typing.

The Australia clade was most closely related to recent North America isolates with a median SNP difference of 43 (IQR 22–60), a genomic distance that overlaps with previous thresholds defining related isolates (Figure 1, panel B). All isolates within the cluster shared the same finetyping and Bexsero Antigen Sequence Typing profile consisting of virulence determinants fHbp, NadA, and NHBA alleles (Figure 2). Although those findings suggest the MenY ST1466 clade was introduced to Australia from North America, the clustering of recent urogenital isolates from Spain within the US clade indicates intercontinental intermixing of isolates and less clear routes of transmission.

**Figure 3:**
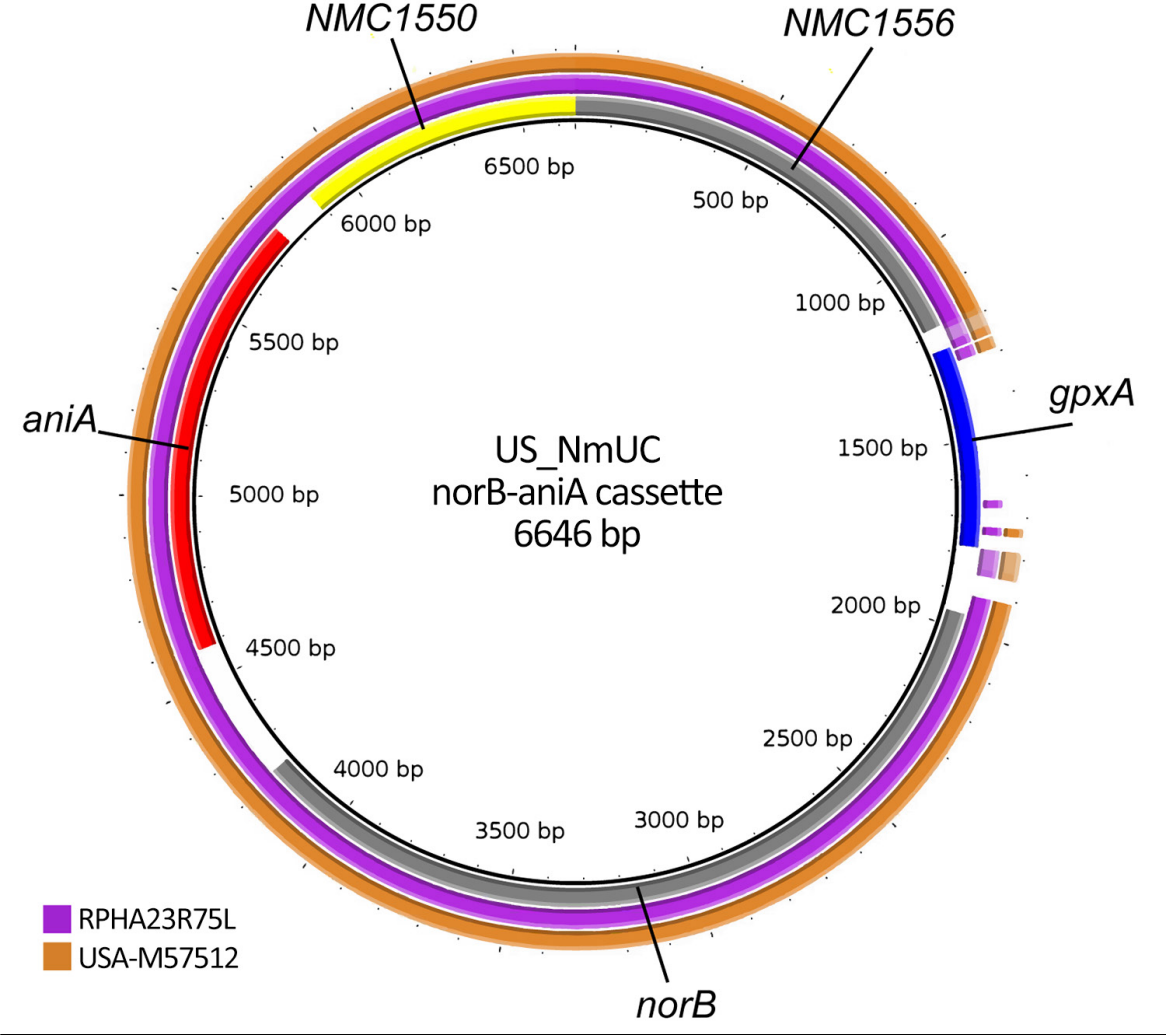
BLAST Ring Image Generator (BRIG) analysis of *Neisseria meningitidis* serogroup Y ST1466 showing sequence from Australia (RPAH23R75L) and sequence provided by the US Centers for Disease Control and Prevention (USA-M57512) relative to the US_NmUC clone across the *norB-aniA* cassette. The locations of 5 genes are shown in the innermost ring. The middle ring depicts alignment for the Australia isolate and the outer for the US isolate.

Having established that the coincident urogenital MenY ST1466 isolates from Australia are closely related genomically to the circulating IMD MenY ST1466 isolates from the United States, we then investigated whether the Australia isolates shared any known genomic features associated with potential urogenital adaptation by comparing the genomes to a another known successfully adapted *N. meningitidis* clone, the nongroupable clonal complex 11 (US_NmUC) clade. We detected no large-scale genomic differences between RPAH23R75L and US_NmUC. We examined in the Australia isolates the genomic regions that within US_NmUC were thought to be important for the organism’s adaptation to the urogenital tract. The capsular biosynthesis genes remained intact for all Australia and non-Australia ST1466 isolates with no evidence of an IS element insertion. A *norB-aniA* gene cassette was present in all the MenY ST1466 sequences. The genomic context of this cassette differed across all ST1466 isolates (represented by the closed genome of RPAH23R75L) and US_NmUC; ST1466 sequences lacked the glutathione peroxidase (*gpxA*) gene (Figure 3). This gene is common to meningococci but was present in only 11 of the 255 ST1466 *N. meningitidis* isolates, suggestive of a gonococcal *norB-aniA* gene cassette recombination event resulting in deletion of gpxA. Although both *norB* and *aniA* were present at the sequence level after alignment, the average pairwise comparison between the MenY ST1466 *norB-aniA* region and the US_NmUC demonstrated ≈39 SNPs difference (4 nonsynonymous mutations), suggesting a different origin and unrelated regions.

Subsequent examination of all the pangenomes including the US_NmUC similarly did not reveal any candidate genes associated with clinical manifestation, which suggests that the observed niche change for MenY ST1466 was not attributable to the adaptative genomic changes previously reported in US_NmUC.

## Discussion

Identifying concurrent outbreaks of urogenital MenY ST-1466 infection in Australia and IMD MenY ST1466 infection in the United States has provided a unique opportunity to collaboratively investigate relatedness of Australia and US isolates. In addition, we were able to explore whether markers of the US_NmUC clade were present to explain the proclivity for the urogenital niche in the current Australian MenY ST1466 isolates. We found that although the IMD and Australia isolates were closely related, the adaptations reported for the US_NmUC clade were not present in either and thus did not explain the current clinical outbreak observed in Australia. Of note, no MenY ST1466 IMD case had been reported in Australia as of September 2024. It remains unclear whether the urogenital outbreak is associated with increased prior pharyngeal carriage. In the only recent study of *N. meningitidis* carriage in Australia, conducted during 2017–2018, one ST1466 isolate was detected from 34,489 participants (*29*). However, it is possible that oropharyngeal carriage of ST1466 became more prevalent after 2018 and acted as the reservoir for urogenital infections. Alternatively, that finding could suggest expansion within a new niche without prior increase in carriage.

We analyzed the available data and identified no specific genomic changes to explain the adaptation of *N. meningitidis* to the urogenital tract. The *norB-aniA* gene cassette was present in all ST1466 sequences. However, experimental work is required to determine whether this cassette is functional. Further, it is uncertain to what extent gene diversity and the absence of the *gpxA* gene may affect the expression of *norB* and *aniA* within the various niches and determine clinical manifestations.

The US_NmUC clade has shown ongoing evolution over time; the number of regions resembling *N. gonorrhoeae* bacteria has increased, consistent with ongoing genetic exchange including antimicrobial resistance determinants between co-localized *Neisseria* species (*9*). Such exchange events may lead to the dissemination of antimicrobial resistance genes within *N. meningitidis*; even though the Australia outbreak may represent a sporadic event, ongoing surveillance of urogenital isolates is required to definitively exclude alternate explanations.

Our investigations provide further evidence that *N. meninigitidis* and *N. gonorrhoeae* can cause overlapping clinical syndromes. However, under the current testing paradigm for urethritis in both Australia and the United States, *N. meningitidis* causing urethritis would be largely undiagnosed because it would not be detected by *N. gonorrhoeae* PCR (*30*). Furthermore, in most settings, isolates from urogenital culture would not be reported because, in many countries, only invasive meningococcal disease is reportable and not noninvasive manifestations such as meningococcal urethritis. The evidence for estimating the risk for urogenital MenY ST1466 to cause invasive disease or, conversely, the propensity for the invasive isolates to inhabit the urogenital niche is lacking. Such evidence would help inform the potential benefits of expanded testing for urogenital *N. meninigitidis* infections and public health actions; they include the utility and recommendations for immunization or prophylaxis for contacts of persons with urogenital meningococcal infections to potentially reduce meningococcal transmission and progression to IMD.

The Australia public health investigation was unable to find any new links, specific sexual behaviors, or at-risk populations associated with the urethritis outbreak. Nevertheless, the investigation raised several questions that warrant further exploration: whether oral sex is the route of transmission of ST1466 meningococcal urethritis; whether the ability of *N. meningitidis* to colonize the urogenital tract results in more successful clones; whether changes in sexual behavior may have contributed to the urethritis cases in Australia; and whether serogroup A, C, W, Y meningococcal vaccination would prevent MenY ST1466 urethritis.

In conclusion, we report a new manifestation of a MenY ST1466 in the urogenital tract and demonstrate that ST1466 urethritis isolates in Australia and invasive isolates in the United States are closely related. Our failure to detect genomic features previously posited to be associated with adaptation to the urogenital niche among ST1466 urethritis isolates demonstrate that further study is needed to understand the mechanisms underlying urogenital adaptation of *N. meningitidis*. However, our findings suggest that the ST1466 *N. meningitidis* isolates circulating in Australia and in the United States can cause both urethritis and IMD, despite the absence of reported ST1466 invasive disease cases to date in Australia. 

Appendix Additional information about isolates of *Neisseria meningitidis* serogroup Y ST-1466 in study of urogenital infections, Australia.
